# Bilateral benign reactive lymphoid hyperplasia of the conjunctiva: a case treated with oral doxycycline and review of the literature

**DOI:** 10.1186/s40662-019-0151-4

**Published:** 2019-09-02

**Authors:** Olga Klavdianou, Georgios Kondylis, Vasileios Georgopoulos, Sotiria Palioura

**Affiliations:** 1 National and Kapodistrian University of Athens School of Medicine, Athens, Greece; 2 Cornea Service, Athens Vision Eye Institute, 328-330 Syngrou Ave, Kallithea, 17673 Athens, Greece

**Keywords:** Conjunctival lymphoma, Conjunctival biopsy, High resolution anterior segment optical coherence tomography, Conjunctival tumor, Conjunctival lymphoid lesion, Atypical lymphoid hyperplasia

## Abstract

**Background:**

To report a case of bilateral benign reactive lymphoid hyperplasia (BRLH) of the conjunctiva treated with oral doxycycline and perform review of the literature evaluating the presentation, treatment and risk of transformation to lymphoma.

**Case presentation:**

A case report is described and review of the literature from January 1975 to January 2019 was performed. A 30-year-old man presented with bilateral enlarging fleshy pink medial canthal conjunctival lesions. Incisional biopsy revealed BRLH. Oral doxycycline was initiated (100 mg two times a day) for a total of 2 months. Both lesions decreased in size significantly at the patient’s two-month follow up visit. The residual lesion in the right eye was excised along with an adjacent pterygium and the patient has been free of recurrence for the past 1.5 years. The lesion in the left eye has remained stable in size after cessation of the oral doxycycline. A total of 235 cases of conjunctival BRLH were identified in our literature search. The mean age at diagnosis was 35.2 years (range, 5 to 91 years). BRLH lesions were unilateral in 75% of patients and bilateral in 25% of them. Seven patients (2.9%) had a concurrent Epstein-Barr virus (EBV) infection at the time of lesion appearance. The most common treatments were surgical excision (155/235 or 65.9%) and corticosteroids (30/235 or 12.7%), while 14% (33/235) of the patients were observed and 4.6% (11/235) received external beam radiotherapy alone. Recurrence occurred in ten patients (10/235 or 4.2%), of whom five had undergone surgical excision alone, two excision followed by external beam radiotherapy, one excision and oral corticosteroids, one radiotherapy alone and one had been treated with topical corticosteroids. Overall, only 2 of the 235 reported cases (0.8%) developed malignancy, one localized to the conjunctiva and one systemic.

**Conclusions:**

Benign reactive lymphoid hyperplasia is one of the lymphoproliferative disorders of the conjunctiva and ocular adnexa. Extensive literature review shows that most cases are treated with surgery, steroids or observation. Oral doxycycline may be considered an alternative non-invasive treatment of BRLH conjunctival lesions. BRLH lesions warrant careful follow up as they can rarely transform into conjunctival or systemic lymphoma.

## Background

Benign reactive lymphoid hyperplasia (BLRH) of the conjunctiva is a rare, lymphoproliferative process that belongs to the broad spectrum of ocular adnexal lymphocytic infiltrative disorders [1–3]. It exhibits a polyclonal proliferation and presents in three different histologic types: follicular, diffuse and sheet-like [4]. The exact etiology and pathogenesis of benign reactive lymphoid hyperplasia (BRLH) remains unknown. However, BRLH is thought to result from a chronic inflammatory response of lymphoid cells to antigenic stimulation [5, 6]. The disorder displays a predilection for the male gender and the most common site of involvement is the nasal conjunctiva [1, 6–11]. Due to the clinical resemblance of BRLH to conjunctival lymphoma and the potential risk of malignant transformation, thorough examination and assessment of such lesions is warranted [1–4, 8, 9, 12, 13]. Various modalities have been used in the treatment of BRLH lesions such as surgical excision, topical, intralesional and/or oral corticosteroids, topical cyclosporine, topical interferon α2b, radiotherapy and observation [1, 2, 4–12, 14–38]. However, there is no established treatment protocol or consensus among experts as to how to manage BRLH lesions. Herein, we report a case of a 30-year-old man with bilateral benign reactive lymphoid hyperplasia of the conjunctiva treated with oral doxycycline and performed a literature review of all reported BRLH cases as to their presentation, treatment, and risk of recurrence and/or transformation to conjunctival or systemic lymphoma.

## Case presentation

**Fig. 1:**
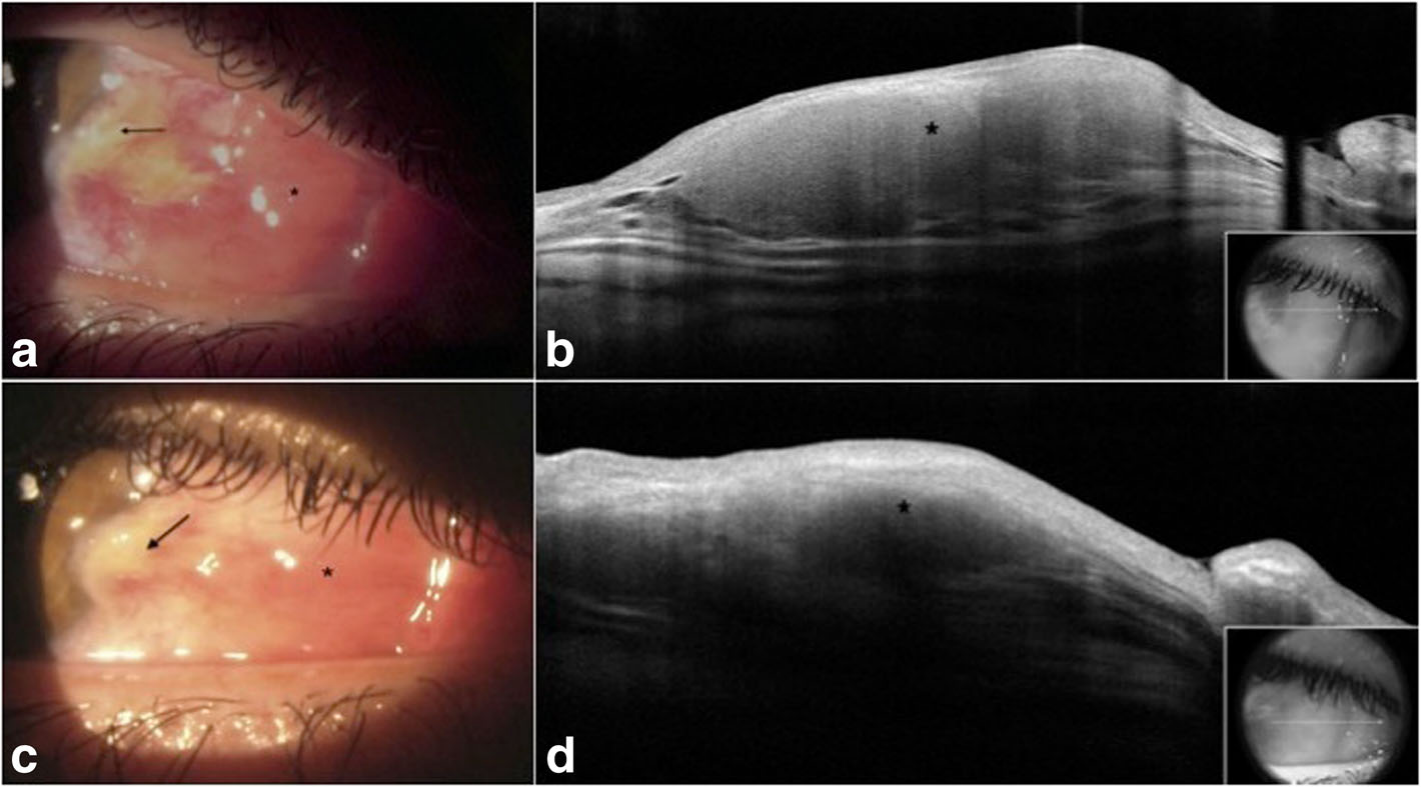
Slit lamp photograph and high resolution anterior segment OCT of the patient’s right eye. **a** A gelatinous, fleshy, firm, pink conjunctival lesion (asterisk) is present in the medial canthal area of the right eye and a pterygium-type lesion with a leukoplakic head encroaching on the cornea adjacent to it (arrow). **b** High resolution anterior segment OCT reveals a homogeneous hyporeflective lesion (asterisk) with thin overlying epithelium in the medial canthal area of the right eye. The inset indicates the level of the scan. **c** Slit lamp photograph of the right eye after 2 months of oral doxycycline 100 mg twice a day. The pterygium (arrow) remains unchanged while the nasal BRLH lesion (asterisk) has decreased in size and appears flatter and smaller. **d** High resolution anterior segment OCT confirms the reduced size of the nasal BRLH lesion (asterisk) after 2 months of oral doxycycline. The inset indicates the level of the scan

**Fig. 2:**
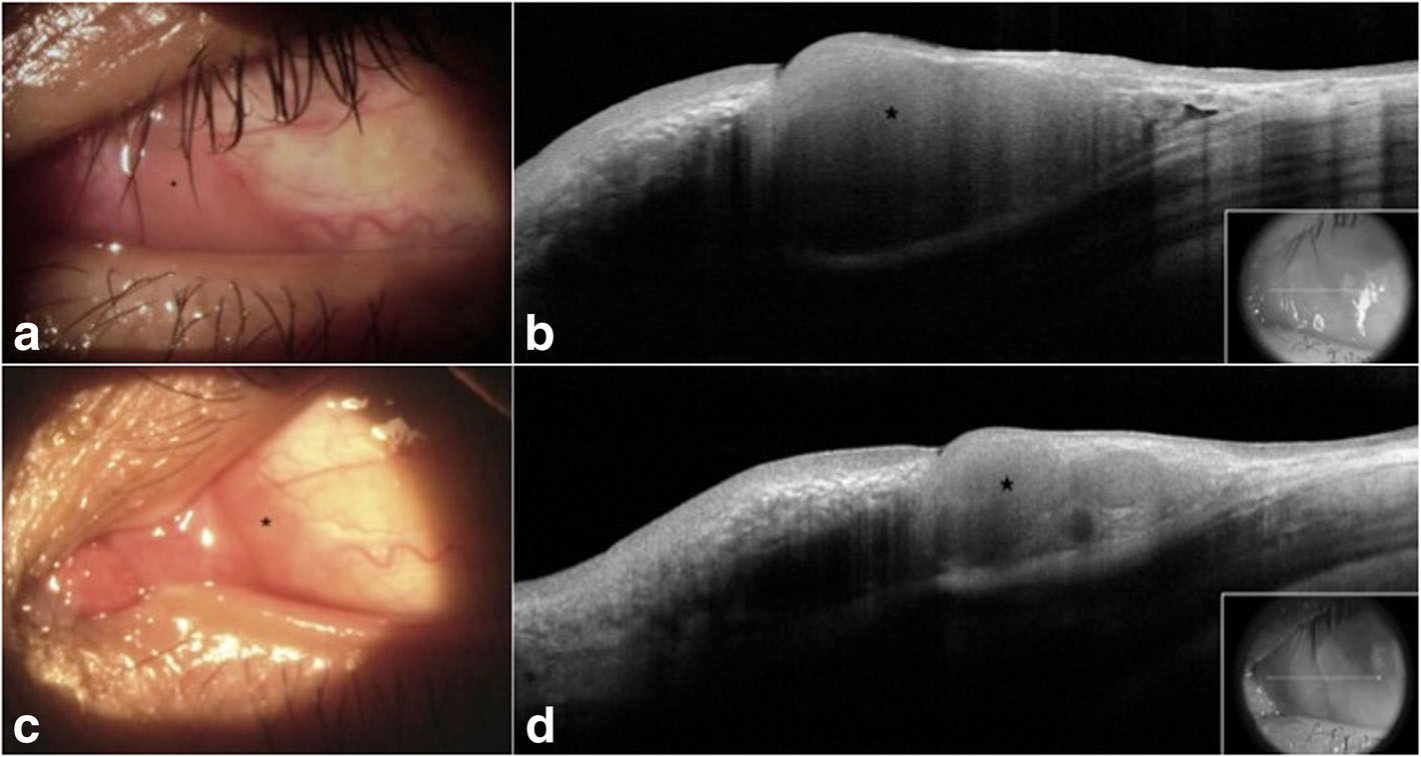
Slit lamp photograph and high resolution anterior segment OCT of the patient’s right eye. **a** A gelatinous, fleshy, firm, pink conjunctival lesion (asterisk) is seen in the medial canthal area of the left eye. **b** High resolution anterior segment OCT reveals a homogeneous hyporeflective lesion (asterisk) with thin overlying epithelium in the medial canthal area of the left eye. The inset indicates the level of the scan. **c** Slit lamp photograph of the left eye after 2 months of oral doxycycline 100 mg twice a day. Similar to the right eye, the nasal BRLH lesion (asterisk) has decreased in size and appears flatter and smaller. **d** High resolution anterior segment OCT confirms the reduced size of the nasal BRLH lesion (asterisk) after 2 months of oral doxycycline. The inset indicates the level of the scan

A 30-year-old man presented to the cornea service at the Athens Vision Eye Institute for evaluation of a new rapidly enlarging lesion in his right eye over the last 6 months. His past medical history was significant for *Escherichia coli* prostatitis 1 year prior to presentation. He had significant sun exposure since childhood, and he worked as a skipper in a sailboat for the last 12 years. His best-corrected vision was 20/20 in both eyes. Upon examination of the right eye, a fleshy pink conjunctival lesion was noted in the medial canthal area (Fig. 1a). In addition, a pterygium-type lesion encroaching on the cornea was noted. Examination of the left eye revealed a smaller fleshy pink conjunctival lesion in the medial canthus (Fig. 2a). High resolution anterior segment optical coherence tomography (OCT) (Optovue Avanti, Fremont, CA, USA) of the bilateral medial canthal lesions revealed homogeneous hyporeflective lesions with thin overlying epithelium (Figs 1b, 2b). Upon further questioning the patient and his family, they reported the presence of the bilateral medial canthal lesions since the patient was a teenager, but the patient had never sought ophthalmic care. The pterygium had been present for 1.5 years and the corresponding OCT revealed mild hyper-reflectivity of the otherwise thin epithelium with underlying subepithelial hyper-reflective “stringy” tissue.

**Fig. 3:**
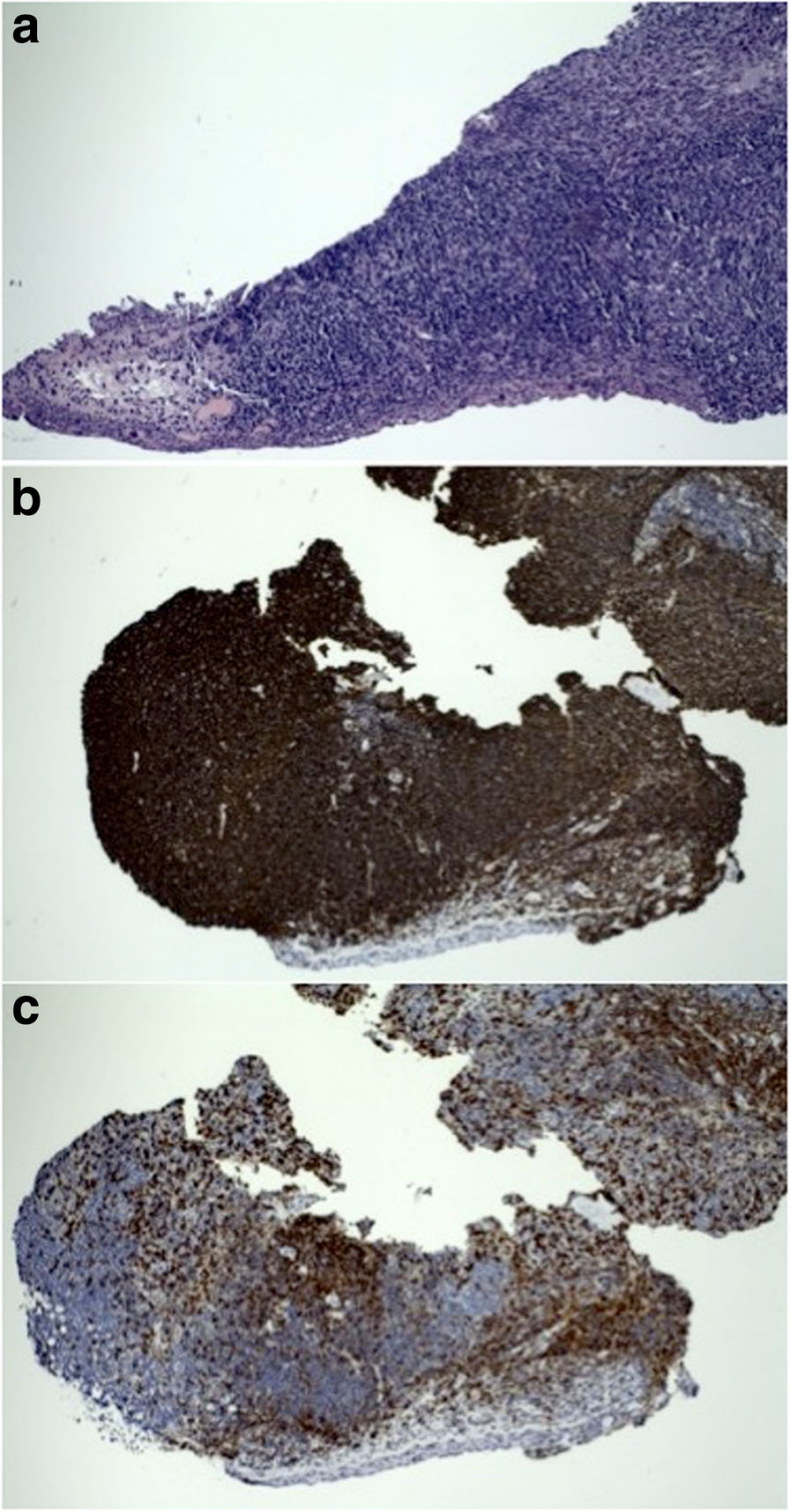
Histopathology of the incisional medical canthal biopsy specimen from the patient’s right eye. **a** Hematoxylin-eosin staining of lymphoid follicles composed of small cells with mitotic figures and tingible body macrophages. (× 100 magnification) (**b**) Dense CD20 staining of B cells. (× 100 magnification) (**c**) CD3 staining of T cells within the follicles and in the interfollicular zones. (× 100 magnification)

Small incisional biopsies (2 mm in diameter) of the medial canthal lesions were undertaken and samples were submitted both in formalin and as fresh tissue for flow cytometry. The slightly atypically appearing pterygium was also biopsied. The patient was started on oral doxycycline 100 mg two times a day. Histopathology of the medial canthal lesions revealed lymphoid follicles of variable size that were composed of a polymorphic population of lymphocytes, dendritic cells and tingible body macrophages. Immunohistochemical staining was positive for CD20, CD3, Bcl-6, CD10 and Ki-67, and negative for Bcl-2 and Cyclin D1 (Fig. 3). Flow cytometry showed a polyclonal population of lymphocytes in both medial canthal lesions. Finally, histopathology of the other lesion in the patient’s right eye revealed elastotic degeneration consistent with pterygium. Oral doxycycline was continued for a total of 2 months. The medial canthal lesions continued to shrink as evidenced both on clinical exam (Figs. 1c, 2c) and high resolution anterior segment OCT (Figs. 1d, 2d). A month later, the patient requested surgical excision of the pterygium for cosmetic reasons and the residual medial canthal lesion in the right eye was also removed. He is free of recurrence of the BRLH for the past 1.5 years. The residual medial canthal lesion in the left eye has not increased in size since stopping the oral doxycycline.

## Literature review

A PubMed search of articles published between January 1975 and January 2019 on the diagnosis and management of benign reactive lymphoid hyperplasia was performed. Searches included a combination of the following terms: “benign reactive lymphoid hyperplasia”, “conjunctival lymphoid hyperplasia”, “conjunctival lymphoma,” “ocular adnexal lymphoma,” conjunctival lymphoproliferative lesion", “conjunctival lymphoid lesion”, “doxycycline”, and “*Chlamydia psittaci.”* The resulting articles and references therein were then reviewed for pertinence.

Literature review revealed 235 reported cases of BRLH in 36 published studies, which are presented in Table 1 [1, 2, 4–12, 14–38]. The mean age at diagnosis of all reported cases was 35.2 years (range, 5 to 91 years), 54% of the patients whose gender was reported were male (100/186), and 46% were female (86/186). BRLH lesions were unilateral in 75% (78/104) of the patients in whom lesion location was reported and bilateral in 25% (26/104) of them. Patients were followed for a mean of 37.3 months (range, 1 month to 14 years). The primary presenting signs and symptoms included irritation and foreign body sensation (15% or 21/138), and redness and swelling (69% or 95/138), while a significant number of patients were asymptomatic (16% or 22/138).

**Table 1:** Review of cases, interventions, and outcomes of benign reactive lymphoid hyperplasia conjunctival lesions

Author	Number of patients	Age in years (*n*)	Laterality (*n*)	Location (*n*)	Treatment modality (*n*)	Recurrence (*n*)	Follow up (months)	Response (*n*)
Parikh et al. (2018) [29]	1	66	Bilateral	Bulbar conjunctiva	Topical cyclosporine 0.05%	None	3	Partial
Moraes et al. (2017) [14]	1	40	Unilateral	Bulbar conjunctiva	Topical corticosteroids	None	36	Complete
Shields et al. (2017) [8] Shields et al. (2004) [9] Shields et al. (2001)^a^ [1]	113	Mean: 43.2 Range: 5.3–86.3 age 0–30, (41) age 31–60, (30) age > 60, (36)	N/A	Fornix (34), Tarsal conjunctiva (6), Plica (15), Caruncle (8), Diffuse (1), Limbal bulbar conjunctiva (13), Extralimbal bulbar conjunctiva (30)	Topical corticosteroids (18), Excision (67), External Beam Radiotherapy (7), Observation (21)	Systemic lymphoma (1)	N/A – survey over 40 years	N/A
Nivean et al. (2017) [19]	1	17	Unilateral	Nasal conjunctiva	Excision followed by oral corticosteroids	None	24	Partial
AlAkeely et al. (2017) [6]	24	Mean: 11.6 Range: 7–17	Unilateral(15) Bilateral (9)	Bulbar conjunctiva, nasally (23) and laterally (1)	Primary excision (17), Topical corticosteroids (4) or topical antihistamines (2) or topical antibiotic (1) followed by excision	1 patient at 1 yr. & 1 patient at 2 yrs. postoperatively, both post-excision	Mean: 49 Range: 12–98	Complete (17), Partial (7)
Vaivanijkul et al. (2017) [37]	1	5	Unilateral	Bulbar conjunctiva	Incisional biopsy & Observation (EBV-related lesion)	None	2	Complete
Brazert et al. (2015) [18]	1	14	Unilateral	Bulbar conjunctiva	Oral methylprednisolone	None	32	Complete
Beykin et al. (2014) [28]	7	Mean: 13.6 Range: 6–21.5	Unilateral (6) Bilateral (1)	Plica semilunaris/caruncle	Excision	None	Mean: 72 Range: 20–168	Complete
Koay et al. (2012) [26]	1	50	Bilateral	Bulbar conjunctiva/Cornea	Excision	None	12	N/A
Herwig et al. (2012) [10]	7	Median: 23 Range: 8–77	Unilateral (4) Bilateral (2)^b^	Caruncle (2), Plica (1), Inferior fornix (1), Nasal conjunctiva adjacent to plica (3)	Excision	None	Range: 12–132	N/A
Al-Mujaini et al. (2012) [27]	2	9 and 14	Unilateral	Nasal conjunctiva close to plica & medial canthus, respectively	Excision	None	Range: 9–10	Complete
Fukuhara et al. (2012)^c^ [12]	1	35	Bilateral	Upper and lower conjunctival fornix	Observation	Conjunctival lymphoma	11	N/A
Ahmed et al. (2011) [16]	1	70	Unilateral	Superomedial conjunctival quadrant	Intralesional steroids (triamcinolone acetonide)	None	N/A	Complete
Lam et al. (2011) [25]	1	13	Bilateral	Bilateral nasal bulbar conjunctiva adjacent to plica	Excision	None	6	Partial
Oh DH et al. (2011) [31]	1	27	Bilateral	Nasal conjunctiva	Anti-VEGF subconjunctival injection (Bevacizumab)	None	12	Complete
Stacy et al. (2010) [4]	6	Median: 40 Range:8–77	Unilateral (5) Bilateral (1)	Inferior formix (1), Inferolateral bulbar conj (1), Superonasal bulbar conj (1), Bilateral medial bulbar conj (1), Plica/caruncular complex (2)	Excision	None	Range: 1–36	N/A
Bagheri et al. (2007) [7]	5	Mean: 14 Range: 6–18	Unilateral (5)	Caruncle lesions (3), plica semilunaris (2)	Excision	None	Mean: 45.8 Range: 2–108	Complete
Finger et al. (2007) [30]	1	33	Bilateral	Superior tarsal conjunctiva and inferior fornix	Topical Interferon 1 MIU/mL	None	6	Partial
Reddy et al. (2006) [20]	1	31	Unilateral	Plica and caruncle	Excision	None	24	Complete
Ioannidis et al. (2005) [15]	1	59	Bilateral	Superotemporal conjunctiva	Topical dexamethasone (Maxitrol)	None	N/A	Partial
Telander et al. (2005) [17]	1	72	Unilateral	Nasal conjunctiva	Subconjunctival steroid injection (triamcinolone acetonide)	None	9	Complete
Rofail et al. (2005) [23]	1	73	Unilateral	Superotemporal conjunctiva	Excision	None	1	N/A
Kim et al. (2005) [24]	1	14	Bilateral	Bulbar conjunctiva, near the medial canthus	Excision	None	24	Complete
Tang et al. (2003) [32]	1	13	Unilateral	Caruncle, plica, and superomedial bulbar conjunctiva	Incisional biopsy & Observation (spontaneous resolution along with patient’s EBV-negative tonsillar enlargement)	None	24	Complete
Mannami et al. (2001) [33]	1	78	Unilateral	N/A	Observation	N/A	Lost to follow up	N/A
Hundsdoerfer et al. (2000) [22]	1	12	Unilateral	Nasal conjunctiva	Incisional biopsy & Observation (EBV-related lesion)	None	8	Complete
Feinberg et al. (2000) [38]	2	19 and 8	Bilateral and Unilateral	Medial canthus/caruncle/plica & Inferior conjunctiva, involving the fornix and extending into the caruncle, respectively	Incisional biopsy & Observation (19 yo patient), Excision (8 yo patient) – Both patients had EBV-related lesions	None	24	Complete
McLeod et al. (1999) [5]	2	12 and 7	Unilateral	Nasal bulbar conjunctiva with involvement of the adjacent semilunar fold & Left caruncle, inferior fornix, respectively	Excision (both patients) followed by oral corticosteroids (7 yo patient)	None	12 and 42	Partial
Coupland et al. (1998) [2]	3	14, 32 and 35	N/A	Conjunctiva	Topical corticosteroids	1 patient after 5 years	Median: 31.3 Range:6.4–125.5	Partial
Urbak et al. (1993) [36]	1	14	Unilateral	Nasal bulbar conjunctiva	Observation (EBV-related lesion)	None	2	Complete
Gardner et al. (1991) [34]	1	38	Unilateral	Superonasal conjunctiva	Observation (EBV-related lesion)	None	12	Complete
Knowles et al. (1990) [21]	9	Median: 61 Range: 17–93	N/A	N/A	Excision	N/A	N/A	N/A
Meisler et al. (1981) [35]	1	11	Unilateral	Upper tarsal conjunctiva	Observation (EBV-related lesion)	None	1	Complete
Sigelman et al. (1978) [11]	33	Median: 55 Range: 9–78	Unilateral (26) Bilateral (7)	Inferior fornix (15), Bulbar conjunctiva (12), Plica/caruncle (4)	Excision (13), Excision & Corticosteroids (1), Excision & External Beam Radiotherapy (13), External Beam Radiotherapy (4), Observation (2)	7 patients (excision: 3, excision & corticosteroids:1, excision & irradiation: 2, irradiation: 1)	72	Complete

*EBV* = Epstein-Barr virus; *N/A* = Information not available or not applicable

^a^ In this series, one patient with BRLH developed systemic lymphoma

^b^ In this study, in both bilateral cases the other eye was already diagnosed with conjunctival lymphoma

^c^ This patient developed extra-nodal marginal zone (EMZL) B cell lymphoma from BRLH in her right eye 11 months after diagnosis of EMZL in her left eye

In terms of lesion location, more than half of the lesions involved the nasal bulbar conjunctiva, one third of them involved the caruncle and plica semilunaris, while the rest of them were located in the fornix and tarsal conjunctiva. Eight patients had enlarged painless pre- or post-auricular lymph nodes at presentation and two presented with enlarged painless submental lymph nodes [5, 6, 11]. Moreover, six patients had concurrent (*n* = 4) or recent (*n* = 2) infectious mononucleosis with generalized lymphadenopathy, fever, tonsillitis and positive Epstein-Barr virus (EBV) serology [22, 34–38]. Other than in the aforementioned six patients, testing for infectious agents in BRLH samples has only been performed in a total of 12 cases (5.2% or 12/229) [6, 10, 38] and has been negative except for one patient with a positive histopathology for EBV latent membrane protein without an obvious clinical history of infectious mononucleosis [38]. In the study by AlAkeely et al., only 5 of the 24 cases were tested by immunohistochemistry for herpes simplex virus (HSV) type 1 (*n* = 3), HSV type 2 (*n* = 3), Cytomegalovirus (CMV) (*n* = 3), *H. pylori* (*n* = 3) and EBV (*n* = 3) due to limited tissue availability and were all negative [6]. In the study by Herwig et al., all six BRLH samples that were tested by PCR for *Chlamydia* species (*C. trachomatis, C. psittaci, C. pneumoniae)* and EBV were also negative [10]. None of the 7 patients with either positive EBV serology or immunohistochemistry developed conjunctival or systemic lymphoma at a median follow up of 8 months (range, 1–24) [22, 34–38]. Overall, only 2 of the 235 reported cases (0.08%) developed conjunctival (*n* = 1, 12) or systemic lymphoma (*n* = 1) [1]. The patient who developed extra-nodal marginal zone (EMZL) B cell conjunctival lymphoma from BRLH in the right eye was a 35-year old woman who was already diagnosed with EMZL in her left eye 11 months prior [12].

In terms of treatment, review of the reported cases of BRLH (Table 1) revealed that the vast majority of the patients (65.9% or 155/235) were treated with surgical excision of the lesion(s), while the second most common approach was observation alone (14% or 33/235). Corticosteroids (topical, intralesional, subconjunctival, and/or oral) and external beam radiotherapy were used in 12.7% (30/235) and 4.6% (11/235) of the patients, respectively. In 5.5% (13/235) of patients, excision was followed by external beam radiotherapy and in 1.3% (3/235) of patients, a combination of oral corticosteroids and surgical excision was used. Apart from the aforementioned traditional therapies, new treatments have emerged over the last few years including subconjunctival injections with anti-VEGF agents [31], topical cyclosporine 0.05% [29], and topical interferon 1 MIU/mL drops [30].

For the 96 patients for whom information on response to treatment were available, 79 (82.3%) experienced a complete response, while 17 (17.7%) only a partial response. (Table 1) Fifty-two of these 96 patients (54.1%) underwent excisional biopsy of the BRLH lesions; two of them had residual lesions, which were observed [5, 25], and two received a post-operative course of oral corticosteroids, which failed to eradicate the lesions [5, 19]. Nine patients (9/96 or 9.3%) were treated with topical corticosteroids; only one patient showed a complete response [14] while the rest of the patients experienced a partial response [2, 6, 15]. Two patients treated with topical antihistamines and one patient treated with a topical antibiotic ointment also had a partial response and their lesions were subsequently excised [6]. In addition, the two patients treated with topical cyclosporine [29] or interferon [30] responded partially as well. Finally, in nine patients (9.3%), the lesions were observed and resolved completely [11, 22, 32, 34–36, 38]; six of these 9 patients had positive EBV serology [22, 34–36, 38] and one had concurrent EBV-negative tonsillar enlargement [32].

Complications from treatment occurred in two cases (0.85% or 2/235). A 14-year old boy with unilateral BRLH that was treated with oral methylprednisolone (1.5 mg/kg/day) for 2 months developed post-steroid acne, which subsided a few weeks after treatment cessation [18]. The second patient developed alopecia while on topical interferon drops, which resolved upon completion of the treatment regimen [30]. Lesion recurrence was observed in 10 patients (4.2%), of whom five (2.1%) had undergone surgical excision alone [6, 11], two (0.8%) excision followed by external beam radiotherapy, one excision and oral corticosteroids (0.4%), one radiotherapy alone (0.4%) and one (0.4%) had been treated with topical corticosteroids [2].

## Discussion

BRLH is a rare, lymphoproliferative disorder of uncertain etiology that usually appears as a salmon-colored subepithelial lesion in the nasal conjunctiva [1, 2, 6]. The differential diagnosis of BRLH lesions includes a wide spectrum of disorders ranging from infections (e.g., Epstein–Barr virus, toxoplasmosis, bartonella) to sarcoidosis and amyloidosis to more aggressive and malignant processes such as atypical lymphoid hyperplasia, conjunctival lymphoma, Ewing sarcoma, Burkitt’s lymphoma, rhabdomyosarcoma, systemic leukemia and/or lymphoma and squamous cell carcinoma. Therefore, proper diagnosis of such lesions calls for a thorough molecular and histopathological assessment to be performed [1–4].

BRLH lesions exhibit reactive lymphoid follicles composed of follicular dendritic cell meshwork, small T-lymphocytes and a polymorphic population of centroblasts and centrocytes of varying sizes. Small mature lymphocytes usually populate the interfollicular zones [4, 6]. These follicles usually present distinct borders, variable size and irregular shape and are divided by wide interfollicular areas with prominent mantle zones [4, 7]. In contrast, neoplastic follicles are more closely packed together, do not vary in size and shape, and their mantle zones may not be evident [4, 7]. Moreover, in the majority of cases, RLH lesions are characterized by polyclonality, as well as the absence of Dutcher bodies and cytologic atypia, nonetheless, these features only favor the diagnosis of the disease and are not pathognomonic [2, 7, 26]. Finally, as far as immunohistochemistry is concerned, the Bcl-2 marker plays a crucial role in differentiating BRLH from follicular lymphoma, as it is usually elevated in follicular lymphoma and negative in BRLH [4, 6].

The pathogenesis of conjunctival BRLH remains unknown. It is thought that chronic antigenic stimulation possibly has a role in tumor appearance [6]. Infectious agents (e.g., HIV, EBV), immunological processes (e.g., rheumatoid arthritis, Sjogren’s syndrome) and ocular allergy have been associated with chronic inflammation of the conjunctiva, inducing the development of BRLH [20, 22, 34–39]. A correlation between infection with *Chlamydia psittaci* and the presence of ocular adnexal lymphoma has been reported in the past, though there is significant geographical variability even within regions of the same country [40–48]. Reported prevalence rates of *C. psittaci* associations with ocular adnexal lymphoma range from 0% in the United States, Japan and the Netherlands to 10–12% in the United Kingdom, China, and Cuba, 47–54% in Austria, Germany and Hungary and 75–87% in South Korea and Italy [41–59]. Interestingly, in cases of conjunctival lymphoma, doxycycline has been effective in lesions that were both Chlamydia positive and Chlamydia negative [44, 58–60]. It has been hypothesized that the doxycycline effect may be due to its anti-inflammatory action rather than an antibiotic one [44, 58–60]. However, as far as conjunctival BRLH is concerned, a correlation with Chlamydia has not been clearly established [10]. In the Italian study by Ferreri et al., 3 of 26 “reactive lymphadenopathy” samples were positive for *C. psittaci* DNA, though it is not specified whether these samples were from conjunctival or from orbital/lacrimal gland lesions [40]. On the other hand, in two studies from Japan, none of the seven reactive lymphoid hyperplasias of the ocular adnexa were positive for *C. psittaci* [51, 52]. Similarly, none of the two conjunctival BRLH cases from the northeastern United States were positive for *C. psittaci* DNA [55]. Consequently, the role of *C. psittaci* in ocular adnexal lymphoproliferative disorders still remains controversial.

Conjunctival BRLH represents the benign end of the spectrum of lymphoproliferative conjunctival lesions, while conjunctival lymphoma is at the malignant end of the spectrum. Differentiation between such malignant and benign lymphoid lesions presents a diagnostic challenge as the majority of patients with either lesion can present with the same constellation of signs and symptoms [1, 2, 27]. Histopathological evaluation with immunohistochemistry, flow cytometry and molecular diagnostics, such as PCR-based immunoglobulin heavy chain (IgH, IgK) gene rearrangement studies can distinguish BRLH from true lymphomas [3, 6, 61, 62].

An additional challenge that BRLH conjunctival lesions pose to the clinician is their potential to develop into conjunctival lymphoma. When compared to BRLH lesions in the orbit, lesions in the conjunctiva have been associated with a lower incidence of transformation to lymphoma [4, 6, 15, 16, 24, 25, 27, 33, 63, 64]. In our review of the literature, only 2 of the 235 reported cases (0.8%) developed malignancy, one localized to the conjunctiva [12] and one systemic [1].

To date, there is no consensus among ocular surface specialists as to the management of conjunctival BRLH lesions. Surgical excision, despite its curative and diagnostic role, is considered by some to be an unnecessary and potentially harmful procedure for a localized and benign disease such as BRLH, especially when concerning pediatric patients [25, 28]. Corticosteroids, despite being an inexpensive solution, are associated with slow regression and poor response especially in residual lesions, with side effects including ocular hypertension and cataract formation [4, 5, 16, 29]. Finally, external beam radiotherapy carries the risk of cataract, dry eye, and rarely, radiation-related retinopathy [5, 27, 29].

In our case, we administered oral doxycycline for 2 months. Doxycycline was chosen because of its track record of being effective both in Chlamydia positive and Chlamydia negative ocular adnexal malignant lymphomas, likely due to its anti-inflammatory action, as discussed previously [44, 58–60]. Since BRLH is also thought to result from chronic antigenic stimulation, we discussed with the patient the off-label use of oral doxycycline. While the patient had a good clinical response in both eyes, the patient desired pterygium excision for cosmetic reasons and thus both lesions were removed from the right eye, and the small residual lesion in the left eye was observed. There has been no lesion recurrence in the right eye and no growth of the residual lesion in the left eye over the last 1.5 years. No adverse effects were observed. To our knowledge, this is the first report on the use of oral doxycycline for BRLH. Doxycycline’s combined antibiotic and anti-inflammatory action, low cost and fewer topical side effects than corticosteroids render it a good alternative in patients with BRLH. It should be noted, though, that the use of oral doxycycline is contraindicated in children under 8 years of age, as well as during pregnancy and breast-feeding. Similar to most cases treated with topical corticosteroids alone, topical antihistamines, cyclosporine or interferon (see Results section and references 2, 6, 14, 15, 29, 30), oral doxycycline resulted in a partial yet sustainable response.

## Conclusions

In summary, we present the first reported case of biopsy-proven BRLH that responded partially to 2 months of oral doxycycline at a dosing of 100 mg twice daily. Similar to conjunctival lymphoma, some cases of BRLH may be responsive to this simple, non-invasive intervention. The prognosis for BRLH is overall favorable based on our review of all published reports, but a small risk of malignant transformation is possible, and thus patients should have long term follow up. Further studies are required to confirm the beneficial role of oral doxycycline in the management of BRLH lesions.

## Data Availability

Data and materials supporting the results reported in the manuscript are available upon request.
